# Association between dairy consumption and the risk of diabetes: A prospective cohort study from the China Health and Nutrition Survey

**DOI:** 10.3389/fnut.2022.997636

**Published:** 2022-09-26

**Authors:** Yucheng Yang, Xiaona Na, Yuandi Xi, Menglu Xi, Haibing Yang, Zhihui Li, Ai Zhao

**Affiliations:** ^1^Vanke School of Public Health, Tsinghua University, Beijing, China; ^2^Institute for Healthy China, Tsinghua University, Beijing, China; ^3^School of Public Health, Capital Medical University, Beijing, China

**Keywords:** dairy consumption, milk, BMI, energy intake, diabetes

## Abstract

Diet is closely related to the risk of diabetes; yet the relationship between dairy consumption and the risk of diabetes is unclear with conflicting evidence from previous studies. This study used data from the Chinese Health and Nutrition Survey to investigate the association between dairy consumption and diabetes. A total of 15,512 adults were included; dairy consumption at each survey was assessed by the 3-day 24-h recall and weighed food record methods, and diabetes occurrence was derived from self-reported information. The association between dairy consumption and diabetes was explored using Cox regression and further stratified with BMI and energy intake. Results indicated that 12,368 (79.7%) participants had no dairy consumption, while 2,179 (14.0%) and 947 (6.1%) consumed dairy at 0.1–100 and >100 g/day, respectively. After adjusting for potential confounders, dairy consumption of 0.1–100 g/day was associated with lower risk of diabetes in all participants (HR 0.53, 95% CI:0.38 −0.74; *P* < 0.001) and males (HR 0.50, 95% CI: 0.31–0.80; *P* = 0.004). According to the restricted cubic splines (RCS), the protective effect on diabetes was significant in the total population with dairy consumption ranging from 25 to 65 g/day (HR <1, *P* = 0.025). In the stratified analysis, consuming 30–80 g/day was associated with reduced diabetes risk among the ≤ 2,000 kcal/day energy intake group (HR <1, *P* = 0.023). In conclusion, dairy consumption was inversely associated with a reduced diabetes risk in Chinese population. Further studies are required to examine the optimal level of dairy consumption for preventing diabetes in the Chinese population.

## Introduction

Diabetes affected the health of approximately 425 million people worldwide in 2015, and it is estimated that this number will increase to 629 million by 2040 (1). In China, the total number of diabetic patients was about 109.6 million, accounting for 10.6% of China's total population (2). The risk factors for diabetes are complex, including obesity, family genetics, lifestyle, and diet (3, 4). Growing evidence has suggested that dietary and nutritional factors, including dairy consumption, may play an important role in the development of diabetes (5, 6).

Dairy is a source of human nutrition and plays an important role in a balanced diet (7, 8). Several epidemiological studies have shown that high consumption of dairy may have protective effects against cancer, coronary heart disease, and all-cause mortality (9–12). Previous studies revealed that dairy can promote weight loss and improve body composition in adults, and it likely contributes to reducing the risk of diabetes (13, 14). However, recent epidemiologic studies on the association between dairy consumption and diabetes have shown conflicting results. A systematic review and meta-analysis showed that higher consumption of dairy was positively associated with diabetes, but this association was found only in Asian and Australian populations, not American and European populations (6). Nevertheless, a study that followed 3,454 middle-aged and elderly Spanish populations found that high consumption of dairy products, especially yogurt, was beneficial in preventing diabetes (15). A study of three large prospective cohort studies in the US evaluated the relationship between dairy consumption and diabetes found that yogurt was associated with a lower risk of diabetes, whereas cheese had the opposite effect (16). Another study on Dutch adults showed that dairy consumption was not associated with diabetes (17).

Different from western countries, dairy products are rarely included in traditional Chinese diets. With the rapid advancement of urbanization, the consumption of dairy products by Chinese residents has been increasing. We hypothesized there is a benefit of dairy consumption on diabetes prevention in the Chinese population. To test this hypothesis, data from a nationwide, prospective cohort study with long-term follow-up was used to explore the associations between dairy consumption and diabetes in the Chinese population. In addition, whether BMI and energy intake affect the relationship between dairy consumption and diabetes or not was further examined.

## Materials and methods

### Study design and participants

This study was a secondary analysis of data collected from an ongoing prospective cohort study named China Health and Nutrition Survey (CHNS) (18), which completed ten rounds from 1989 to 2015. All data for this study are publicly available on the website http://www.cpc.unc.edu/projects/china. All participants signed informed consent before enrolling in the study. This study was approved by the National Institute of Nutrition and Food Safety (China) and the institutional review committees of the University of North Carolina (USA).

**Figure 1:**
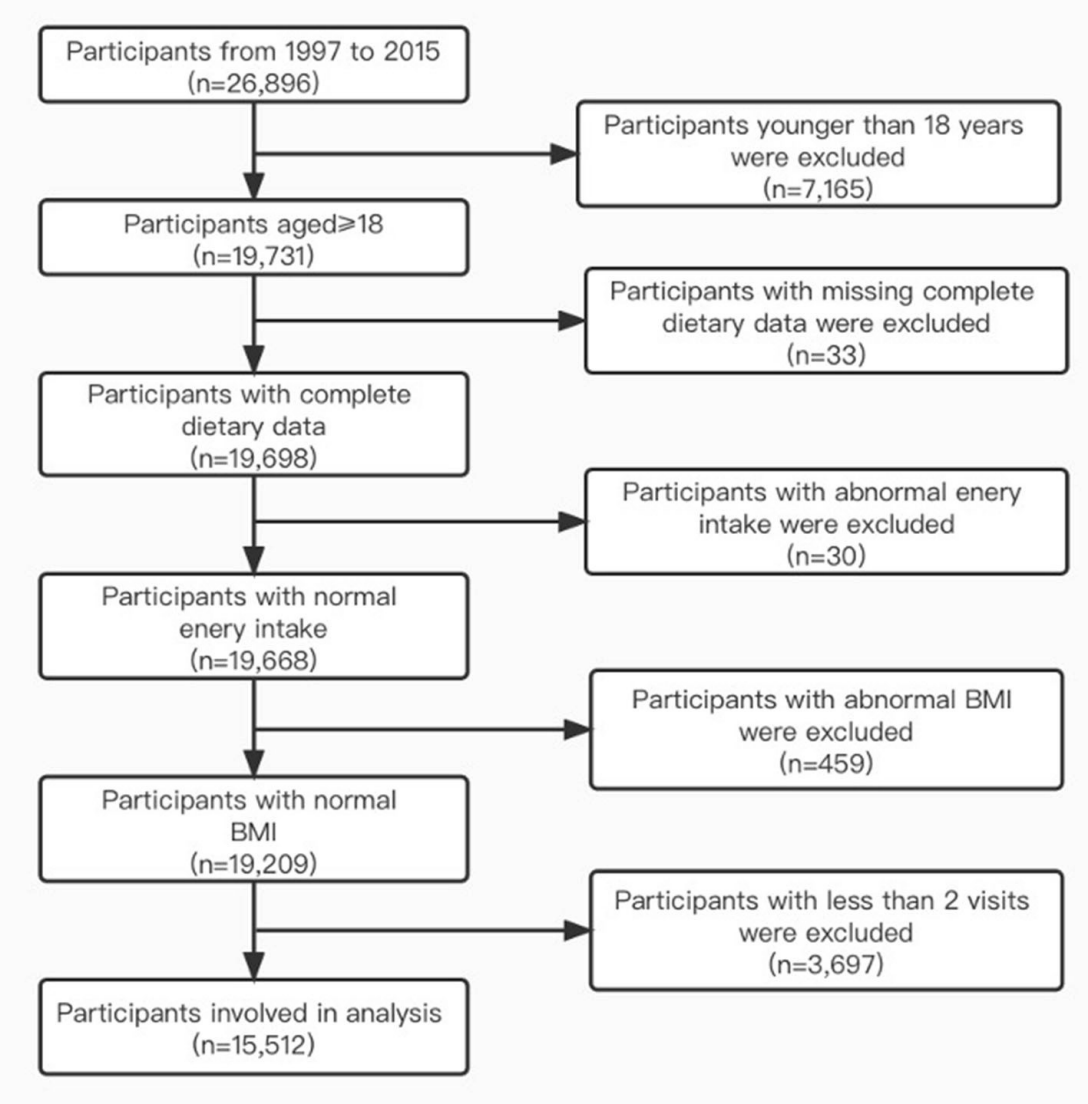
Flow chart of participant selection.

Since the dietary data codes for 1989 and 1993 were not identifiable from the CHNS database, in this study, we used the data collected from this longitudinal open cohort study in 1997, 2000, 2004, 2006, 2009, 2011, and 2015. Because the year of participants entering the cohort was varied, the time each participant entered the cohort was the individual's baseline. By the end of 2015, there were 26,896 available participants in the CHNS, who had not been diagnosed with diabetes at baseline. We excluded participants younger than 18 years old at baseline (*n* = 7,165), participants with missing complete dietary data (*n* = 33), participants who had a total energy intake <500 or >4,500 kcal/day (*n* = 30), participants with a body mass index (BMI) > 40kg/m^2^ (*n* = 459), and participants with fewer than two visits in this study (*n* = 3,697). Finally, we included 15,512 adults in our study from 1997 to 2015 (Figure 1).

### Definition of diabetes

In our study, the outcome variable was primarily defined as self-reported diabetes. Diabetes was identified by one question in the questionnaire: ‘Has your doctor ever told you that you have diabetes?' If the participant answered: ‘Yes', then proceeded to ask: ‘How old were you when you were diagnosed with diabetes?'

### Covariates at baseline

The baseline questionnaire included questions on socio-demographic, lifestyle factors, and health-related information. Socio-demographic characteristics were specifically included as follows: age, gender, income, education level, and living area; lifestyle factors including smoking status and physical activity; health-related information including BMI and disease history. BMI was calculated as weight divided by height squared (kg/m^2^). Physical activity included occupational physical activity, transportation physical activity, leisure time physical activity, and housework physical activity (19). Each participant's metabolic equivalent time of physical activity (MET hours/week) was calculated in weeks based on the allocation of metabolic equivalents (MET). Disease history was defined as having at least one of the following diseases: hypertension, myocardial infarction, stroke, and cancer.

### Dietary consumption information

The three-day (including two working days and one weekend day) 24-h recall was used to collect dietary data at the individual level. Participants were required to recall food intake except for oil, salt, and condiments within 24 h before the survey. The consumption of oil, salt, and condiments were investigated at the household level by weighing accounting method and further divided into individuals according to the person to family energy consumption ratio (20). The Chinese Food Composition Table (1991 (21), 2004 (22), and 2009 (23) editions) was referenced to calculate the energy and nutrient intake of individuals. Dairy consumption was defined as the total consumption of liquid milk, powdered milk, yogurt, and other dairy products such as cream and cheese. In this study, the average consumption of dairy from 1997 to 2015 was calculated based on a 3-day 24-h recall. Dairy consumption was divided into three groups: no consumption, 0.1–100 g/day, and >100 g/day.

### Statistical analysis

Statistical analyses were performed using R version 4.1.1, a two side *P*-value < 0.05 considered to be statistically significant. The median and 25th and 75th quartiles were used to describe the non-normally distributed numerical variables, and frequency and percentage were used to describe the categorical variables. Chi-squares (χ^2^) tests and one-way analysis of variance (ANOVA) were used to compare baseline characteristics of the total study population according to the dairy consumption level. The association between dairy consumption and diabetes was explored using Cox proportion hazard regression and further stratified by gender. The Cox proportion hazard regression included three models: Model 1 didn't adjust for any confounder; Model 2 adjusted for age, gender, education level, living area, income, smoking status, BMI, disease history, and physical activity; Model 3 further adjusted for intake of vegetables, fruit, meat, alcohol, carbohydrate, and energy based on Model 2. The non-linear association between dairy consumption and diabetes was also evaluated using Cox models with restricted cubic splines (RCS). We further examined the stratified analysis to identify the potential effects among participants with different BMI and energy intake levels. To verify the robustness of our research data, we did a sensitivity analysis to exclude participants who died within 2 years of the baseline survey.

## Result

### Baseline characteristics of the participants

Table 1 shows the baseline characteristics of the participants. Among the 15,512 participants included in this study, the mean age of participants was 43.4 years; 7,376 (47.6%) were males. In total, 12,368 (79.7%) of the participants had no dairy consumption in eight rounds of the survey from 1997 to 2015. Of participants with dairy consumption, 2,179 (14.0%) and 947 (6.1%) consumed 0.1–100 g/day and >100 g/day dairy, respectively. Participants with higher consumption were older, had smoked, and had lower levels of education and income; meanwhile, BMI and physical activity levels tended to increase with dairy consumption. In addition, statistical differences in age, gender, living area, and disease history were also significant.

**Table 1:** Baseline characteristics of the participants by levels of dairy consumption.

	**Dairy consumption**			***P***
	**No consumption** ***N* = 12,386**	**0.1–100g/day** ***N* = 2,179**	**>100g/day** ***N* = 947**	
Age, M (P25, P75)	41.0 [31.0,53.0]	43.0 [33.0,54.0]	50.0 [37.0,61.0]	<0.001
Gender, *n* (%)				<0.001
Male	6,008 (81.5%)	976(13.2%)	392 (5.31%)	
Female	6,378 (78.4%)	1203 (14.8%)	555 (6.82%)	
Education level, *n* (%)				<0.001
Junior high school or below	9,820 (86.7%)	1,134 (10.0%)	372 (3.28%)	
Senior high school or vocational school	2,071 (65.9%)	747 (23.8%)	325 (10.3%)	
University or above	484 (46.9%)	298 (28.9%)	250 (24.2%)	
Living area, *n* (%)				<0.001
East	4,022 (68.2%)	1,152 (19.5%)	725 (12.3%)	
Central	4,937 (85.6%)	746 (12.9%)	85 (1.47%)	
West	3,427 (89.1%)	281 (7.31%)	137 (3.56%)	
Income, yuan, *n* (%)				<0.001
<30,000	10,834 (81.2%)	1,871 (14.0%)	631 (4.73%)	
≥30,000	706 (61.3%)	216 (17.4%)	264 (21.3%)	
Smoking status, *n* (%)				<0.001
Never	8,305 (78.5%)	1,538 (14.5%)	736 (6.96%)	
Yes	4,079 (82.8%)	640 (13.0%)	2,110(4.26%)	
BMI, kg/m2, M (P25, P75)	22.3 [20.4,24.7]	23.0 [21.0,25.3]	23.4 [21.2,25.6]	<0.001
Physical activity, MET-hour/week, M (P25, P75)	130 [0.00,613]	180 [11.6,577]	501 [120,1,293]	<0.001
Disease history a, *n* (%)				<0.001
No	9,530 (81.0%)	12,569 (13.3%)	672 (5.71%)	
Yes	2,856 (76.3%)	610 (16.3%)	275 (7.35%)	

^*a*^Disease history included hypertension, myocardial infarction, stroke, and cancer.

### Dietary intake characteristics of the participants

Table 2 shows the dietary intake characteristics of the participants. Foods and nutrients consumption among the three groups were statistically significant. Compared with the no consumption group, participants with the highest dairy consumption had a higher intake of meat, eggs, protein, and calcium, but lower consumption of cereals, carbohydrates, and energy. Furthermore, participants with higher dairy consumption generally had a lower intake of fruits, fats, and dairy calcium, but a higher intake of vegetables.

**Table 2:** Dietary intake characteristics of the participants by levels of dairy consumption. Median (P25, P75).

	**No consumption *N* = 12,368**	**0.1–100g/day** ***N* = 2,179**	**>100g/day** ***N* = 947**	***P***
**Food consumption**
Vegetables intake, g/day	215 [138,300]	205 [144,279]	240 [171,327]	<0.001
Fruit intake, g/day	0.00 [0.00,37.2]	46.7 [4.44,100]	100 [33.3,180]	<0.001
Meat intake, g/day	38.3 [10.0,74.4]	56.7 [31.5,91.6]	70.0 [36.7,110]	<0.001
Cereal intake, g/day	275 [192,367]	245 [185,314]	250 [193,327]	<0.001
Egg intake, g/day	13.3 [0.67,26.7]	25.7 [12.8,40.7]	36.8 [20.0,60.0]	<0.001
Alcohol intake, g/week	0.00 [0.00,579]	0.00 [0.00,600]	0.00 [0.00,181]	<0.001
**Energy and nutrient consumption**
Carbohydrate intake, g/day	300 [242,361]	258 [216,300]	223 [174,275]	<0.001
Fat intake, g/day	65.5 [49.6,83.9]	77.1 [61.8,94.7]	75.2 [57.8,95.4]	<0.001
Protein intake, g/day	63.4 [53.2,74.6]	67.8 [57.9,79.5]	71.6 [58.5,85.2]	<0.001
Energy intake, kcal/day	2,098 [1,772, 2,444]	2,037 [1,751, 2,337]	1,904 [1,577, 2,216]	<0.001
Total calcium intake, g/day	343 [273,435]	414 [338,505]	579 [470,731]	<0.001
Dairy calcium intake, g/day	0.00 [0.00,0.00]	39.3 [16.3,68.8]	173 [136,236]	<0.001

### Association between dairy consumption and risk of diabetes

Table 3 shows the associations between dairy consumption and diabetes. During a median follow-up of 9.0 years, a total of 390 (2.5%) diabetes cases were newly diagnosed. No association was found between dairy consumption and diabetes in the crude model. After multivariable adjustment (Model 2), an inverse association was observed between consuming 0.1–100 g/day of dairy and diabetes (HR 0.53, 95% CI: 0.38–0.74; *P* < 0.001); a similar relationship was observed in Model 3 (HR 0.60, 95% CI: 0.43–0.86; *P* = 0.005) in which food and nutrient consumption was further adjusted. However, among those who consumed >100 g/day of dairy, dairy consumption was not associated with the risk of diabetes.

**Table 3:** HRs (95% CIs) of diabetes risk according to dairy consumption.

	**No consumption**	**0.1–100g/day**	***P***	**>100g/day**	***P***
**All participants**				
Case/*n*	327/12,386	44/2,179		19/947	
Model 1	1.00 (Reference)	0.76 [0.56, 1.05]	0.095	1.52 [0.96, 2.43]	0.076
Model 2	1.00 (Reference)	0.53 [0.38, 0.74]	<0.001	0.96 [0.57, 1.57]	0.865
Model 3	1.00 (Reference)	0.62 [0.44, 0.88]	0.008	1.516 [0.88, 2.61]	0.132
**Male**
Case/*n*	164/6,008	22/976		8/392	
Model 1	1.00 (Reference)	0.82 [0.52, 1.28]	0.376	1.47 [0.72, 3.01]	0.289
Model 2	1.00 (Reference)	0.50 [0.31, 0.80]	0.004	0.82 [0.39, 1.75]	0.610
Model 3	1.00 (Reference)	0.61 [0.37, 0.98]	0.044	1.32 [0.60, 2.93]	0.488
**Female**
Case/*n*	163/6,378	22/1,203		11/555	
Model 1	1.00 (Reference)	0.72 [0.46, 1.12]	0.149	1.58 [0.85, 2.93]	0.146
Model 2	1.00 (Reference)	0.55 [0.34, 0.89]	0.146	1.10 [0.55, 2.18]	0.791
Model 3	1.00 (Reference)	0.66 [0.11, 1.09]	0.109	1.57 [0.74, 3.37]	0.290

Model 1 had no adjustment for confounders; Model 2 adjusted for age, gender, education level, living area, income, smoking status, BMI, disease history, and physical activity; Model 3 was based on Model 2 and further adjusted for intake of vegetables, fruit, meat, alcohol, carbohydrate, and energy.

In the gender stratified analysis, there were no associations between dairy consumption and diabetes in females. However, after multivariable adjustment (Model 2), consuming 0.1–100 g/day of dairy was related to a lower risk of diabetes in comparison to no dairy consumption in males (HR 0.50, 95% CI: 0.31–0.80; *P* = 0.004), and the similar relationship was observed in Model 3 (HR 0.61, 95% CI: 0.37–0.98; *P* = 0.044).

###  Non-linear association between dairy consumption and risk of diabetes

**Figure 2:**
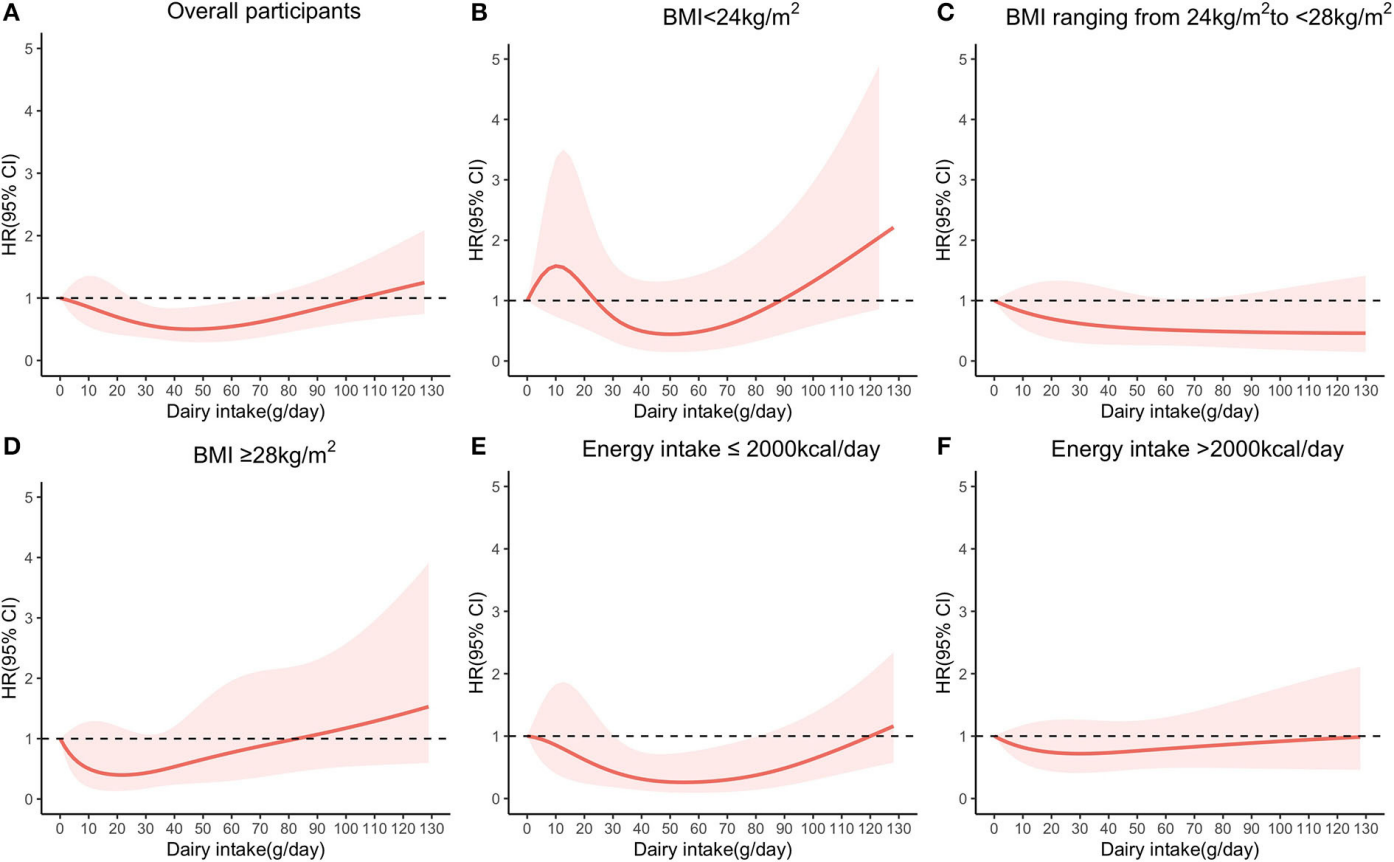
Restricted cubic spline plots to evaluate relationships between dairy consumption and diabetes among **(A)** overall participants; **(B)** participants with BMI <24 kg/m^2^; **(C)** participants with BMI ranging from 24 to <28 kg/m^2^; **(D)** participants with BMI ≥28 kg/m^2^. According to the median energy intake of 2,094.4 kcal/day, we stratified energy intake as **(E)** participants with energy intake of ≤ 2,000 kcal/day and **(F)** participants with energy intake of >2,000 kcal/day. HR and 95% CI were adjusted for age, gender, education level, living area, income, smoking status, BMI, disease history, physical activity, vegetable intake, fruit intake, meat intake, carbohydrate intake, and energy intake. BMI and energy intake were not adjusted in the corresponding stratified model.

The non-linear relationships between dairy consumption and diabetes among the overall population and certain populations are shown in Figure 2. Among the total participants, dairy consumption of about 25–65 g/day was associated with a decreased risk of diabetes (HR <1, *P* = 0.025), then the risk of diabetes increased with increasing consumption. In the subgroup analysis, there was no association between dairy consumption and the incidence of type 2 diabetes at different BMI levels. Among those with an energy intake of ≤ 2,000 kcal/day, dairy consumption of about 30–80 g/day was associated with a decreased risk of diabetes (HR <1, *P* = 0.023), but no association was observed in the higher energy intake groups.

### Sensitivity analysis

In the sensitivity analysis (Supplementary Table 1), we excluded participants who died within 2 years of the baseline survey. Among all participants and male participants, the associations between dairy consumption on average and diabetes were similar to the results shown in Table 3. However, for female participants, compared with the no consumption group, there was an additional positive association between consuming 0.1–100 g/day of dairy and diabetes (HR 0.55, 95% CI: 0.34–0.88; *P* = 0.013) in Model 2.

## Discussion

In the current study, we investigated the association between dairy consumption and diabetes among Chinese adults. We found that, in 15,512 individuals, there was a non-linear association between dairy consumption and diabetes. Those who consumed 0.1–100 g/day of dairy were positively associated with diabetes compared to the no dairy consumption group. The protective effect on diabetes was most significant in the population with dairy consumption ranging from 25 to 65 g/day.

Our finding on the positive association between dairy consumption and risk of diabetes was supported by previous studies in Western populations, such as European Prospective Investigation into Cancer and Nutrition (EPIC) and Mediterranean populations (24–26). However, several studies have reported that higher dairy consumption can reduce the risk of diabetes (27, 28), this is contrary to our findings. This inconsistency may be attributed to differences in Chinese and Western dietary habits. Among the Chinese population, dairy consumption is generally low, and limited types of dairy are consumed. In this study, dairy products mainly included liquid milk, milk powder, yogurt, and other dairy products. But in the above studies, the definitions of dairy products were different: some included milk beverages or cream and ice cream (24, 26). On the other hand, the inconsistent results may be because of the potential confounders considered in different studies. In the current study, after further adjusting the baseline and dietary variables (model 3), the protective associations we expected were not observed, which may support the notion that the nutrients in dairy are the key protective factor regarding diabetes. In addition, it is generally believed that dairy consumption is associated with a healthier diet and a better lifestyle (29); the disappearance of the protective effect of dairy consumption on diabetes after adjusting for dietary factors may also be related.

**Figure 3:**
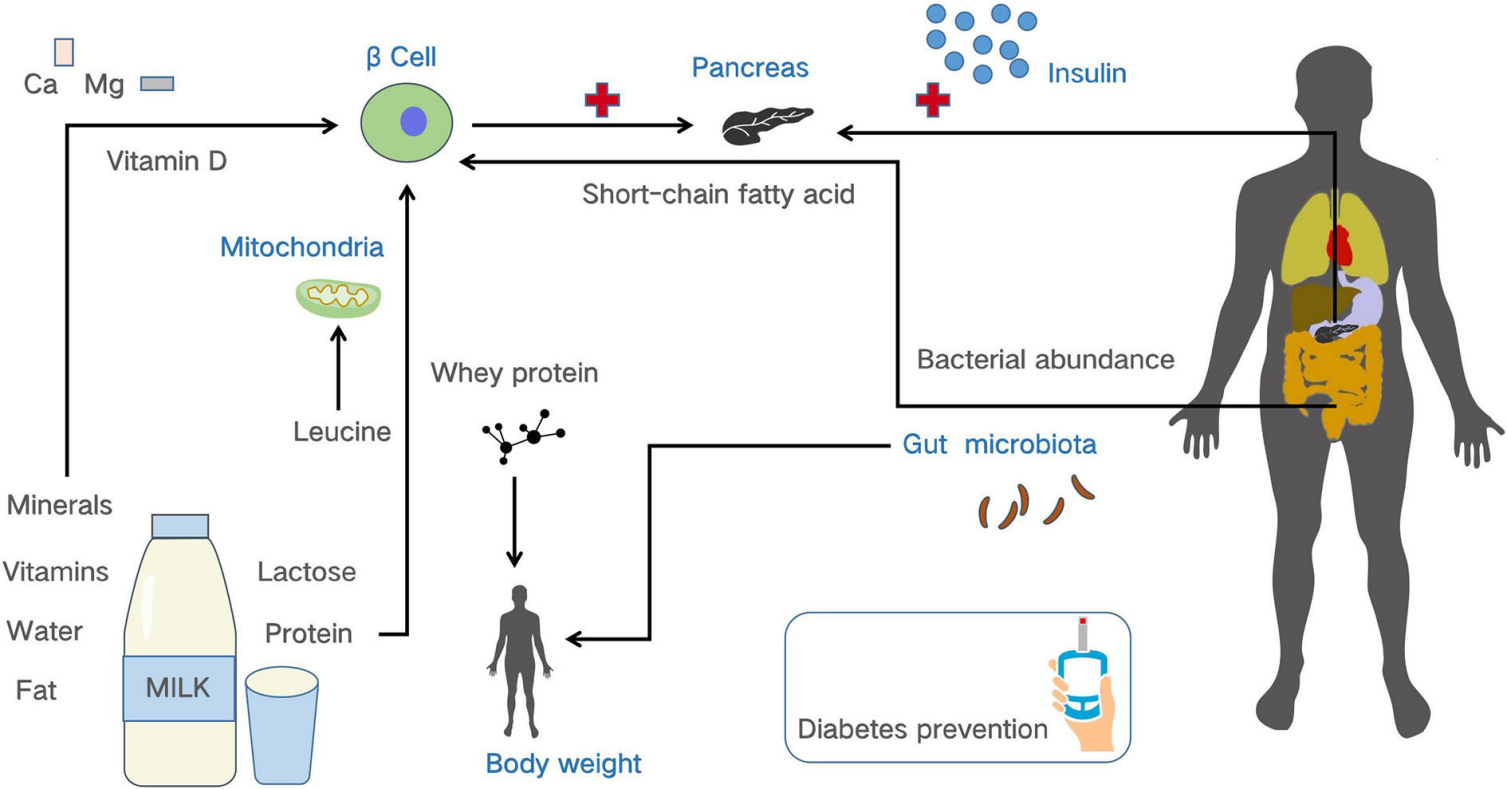
The possible mechanisms of dairy components in preventing diabetes (30–42).

The protective mechanism of dairy consumption on diabetes can be explained from the following aspects (Figure 3) (30–42). Firstly, dairy contains high amounts of essential micronutrients for our body, such as vitamin D, calcium, and magnesium, which can affect insulin sensitivity. Vitamin D may benefit pancreatic β cell function, and calcium and magnesium could both improve insulin sensitivity, thereby helping to improve insulin secretion, which is conducive to the control of blood glucose levels (30, 31). Secondly, whey protein and casein are the two most abundant proteins in dairy, of which whey protein can increase the concentration of amino acids after digestion and promote insulin secretion (32). Whey protein can also improve energy intake balance and promote weight loss, thereby affecting the development of diabetes (33, 34). Furthermore, leucine is an essential amino acid in protein, recent evidence points to the possibility that mitochondrial dysfunction may lead to insulin resistance, and the leucine of dairy could promote mitochondrial production and increase the body's antioxidant capacity, which provides a possible way to relieve insulin resistance (35–38). Thirdly, the oligosaccharides contained in dairy can promote the growth of bifidobacterium growth in the gut, and bifidobacterium has the effect of inhibiting obesity (39), which is an important cause of diabetes. Additionally, in a rodent study, mice fed a high-dairy diet reduced the number of obesity-causing bacteria such as Desulfovibrio and Bacteroidetes (40), which can affect the development of diabetes. Consumption of dairy products can also increase the number of butyrate-producing bacteria in the Firmicutes phylum. Butyrate is an anti-inflammatory short-chain fatty acid that regulates gut health and improves beta cell function, thereby reducing insulin resistance (41, 42).

A dose-response meta-analysis suggested a linear inverse association between low-fat dairy and diabetes (43). However, in our study, we found a non-linear relationship between dairy consumption and diabetes. The protective effect was only observed in those who consumed 0.1–100 g/day of dairy in adjusted Model 2; when participants' dairy consumption exceeded 100 g/day, no association was observed. More particularly, the RCS Cox regression in the current study further revealed that the consumption of 25–65 g/day of dairy might be associated with reduced diabetes risk. Previous research has pointed out that dairy may have adverse health effects when consumed in excess (12, 44, 45). This association may be due to the increased risk of hormone-dependent diseases caused by animal oestrogens in dairy (45, 46). A previous study in India also demonstrated that ingestion of excessive dairy could reduce the insulin sensitivity of individuals, thereby affecting the risk of diabetes (47). Moreover, higher dairy consumption is accompanied by greater energy intake, which increases body weight and therefore may increase the risk of developing diabetes (48). In the stratified analysis, we also found a significant inverse association between dairy consumption and diabetes risk in the ≤ 2,000 kcal/day energy intake group, which supports the previous view that there is a close relationship among dairy consumption, energy intake, and the risk of diabetes (49).

It should be noted that the recommended amounts for dairy consumption in the dietary guidelines of the US and Australia are 710 ml and 500 g, respectively (50, 51). According to the Chinese Dietary Guidelines (2016) (52), the recommended consumption of dairy products is 300 g/day, while the latest Chinese Dietary Guidelines (2022) revised the recommended consumption of dairy products to 300–500 g (53). According to the RCS analysis, the risk of diabetes was reduced only with 25–65 g/day of dairy consumption in the total population. Similar to the results of a previous study conducted in a Chinese population, higher dairy consumption was not necessarily better (12). Although according to the current Chinese study, there is still a great gap between dairy consumption (average consumption is 12 g/d) and recommendation (53), it is time to seriously consider the most optimal consumption level of dairy for varied populations. The recommended consumption of dairy for the Chinese population may require further verification.

In the stratified analyses, we found that the association between dairy consumption and diabetes also differed by gender. Among males, those who consumed 0.1–100 g/day of dairy had a significantly lower risk of diabetes in adjusted Models, which was similar results to the Health Examinees (HEXA) study among Korean adults (54). However, a study of 2,375 males aged 45–59 years found no association between the consumption of dairy and the risk of diabetes (55). Interestingly, a cross-sectional study in Qingdao, China found that an inverse relationship between diabetes prevalence and dairy consumption in females (56), but not in males, which is the exact opposite of our findings. Prolactin is involved in regulating glucose homeostasis and insulin sensitivity (57). For females under the action of body hormones, the hormonal effects of dairy may not be significant; however, this active substance may affect the risk of diabetes in males. The gender differences due to different socio-cultural characteristics and dietary structures also differ between males and females. A prospective study among black women in the U.S. found no link between yogurt and diabetes because the association was attenuated after controlling for healthy eating behaviors associated with yogurt consumption (58). However, our study did not measure hormone levels in each participant and dairy, and there is insufficient evidence to determine whether dairy consumption changed insulin levels. In conclusion, further research is needed to clarify the distinctive effect of dairy consumption on the risk of diabetes in different genders.

Obesity is closely related to chronic non-communicable diseases, including diabetes (48, 59). Several studies have revealed the certain role of obesity in the chain of association between dairy consumption and obesity. Recent studies has found that neither whole milk nor low-fat milk was associated with weight change, and cheese consumption may be beneficial for lowering BMI (60). A prospective cohort study of postmenopausal women found an inverse relationship between low dairy consumption and diabetes; this relationship was more pronounced in the high BMI group (61). One animal study revealed the possible mechanism that dairy products can modulate the gut microbiota and circulate metabolites, resulting in weight loss (40). However, there is no evidence in our study that dairy consumption was associated with the risk of diabetes in different BMI categories (<24, from 24 to <28, ≥28 kg/m^2^), which could necessitate further investigation to verify.

## Limitation

To our knowledge, this is the first report that there is a non-linear association between dairy consumption and diabetes in the Chinese population. However, several limitations of the current study should be noted. First, the determination of the outcome variable was based on self-reported diabetes rather than biochemical indicators. In CHNS, the blood samples of the participants were only collected in 2009 and limited biomarkers were measured, which does not allow us to diagnose diabetes, hence the incidence of diabetes among the overall population may be underestimated. Moreover, we did not distinguish between types of diabetes, and the incidence of specific types of diabetes and its association with dairy consumption could not be observed. Second, due to the low consumption of dairy in the Chinese population, we did not distinguish between dairy types when analyzing the relationship between dairy consumption and diabetes, which may lead to inconsistencies between our findings and those of previous studies. Previous research pointed out that the relationship between dairy and diabetes appears to be related to dairy type (60). Besides, the proportions of micronutrients in different processed dairy products are varied (8, 62), therefore the mis-classification bias may not be ruled out in the current study. Third, dietary intake in this study was estimated from three dietary recall questionnaires, which may not represent long-term intake well and may suffer from recall bias. Furthermore, with the development of the economy, the consumption of dairy increases, and the dynamic changes of dairy consumption with age may also contribute to the development of diabetes. Finally, although our models adjusted for many covariates and the sensitivity analysis showed that the results were stable, the relationship between dairy consumption and diabetes might be ascribed to other potential residual confounding bias, such as genetic backgrounds of diabetes and other environmental factors.

## Conclusion

In conclusion, we found that moderate consumption of dairy can help reduce the risk of diabetes in the Chinese population. As the effects of dairy consumption on the health of different populations are not uniform, our study provides new insights that require careful consideration of the optimal consumption amount of dairy for diabetes prevention.

## Recommendation

In view of the findings of this current study and the dietary habits of the Chinese population, we suggest that moderate consumption of dairy in daily life in the Chinese population may be an effective lifestyle intervention for the prevention of diabetes. In addition, the energy from dairy should be taken into consideration, and balanced energy in the daily diet should be achieved.

## Data availability statement

The datasets presented in this study can be found in online repositories. The names of the repository/repositories and accession number(s) can be found in the article/Supplementary material.

## Ethics statement

The studies involving human participants were reviewed and approved by the National Institute of Nutrition and Food Safety (China) and the institutional review committees of the University of North Carolina (USA). The patients/participants provided their written informed consent to participate in this study.

## Author contributions

YY: conceived the idea. YY and XN: performed the statistical analyses and designed the study. YY, ZL, and AZ: wrote the manuscript. YX, MX, and HY: participated in the discussion and revised the manuscript. All authors read and approved the final manuscript. All authors contributed to the article and approved the submitted version.

## Funding

The CHNS project was funded by many organizations. Major funding for the survey and data dissemination from 1991 to 2004 came from the (NIH) (P01-HD28076 and HD30880). Additional funding was from the NIH (HD39183), the Carolina Population Center, the Ford Foundation, the National Science Foundation (INT-9215399), the National Institute of Nutrition and Food Safety, and the Chinese Center for Disease Control and Prevention. In addition, the current study was supported by the Sanming Project of Medicine in Shenzhen (SZSM202111001).

## Conflict of interest

The authors declare that the research was conducted in the absence of any commercial or financial relationships that could be construed as a potential conflict of interest.

## Publisher's note

All claims expressed in this article are solely those of the authors and do not necessarily represent those of their affiliated organizations, or those of the publisher, the editors and the reviewers. Any product that may be evaluated in this article, or claim that may be made by its manufacturer, is not guaranteed or endorsed by the publisher.
