# Supplementary material for: Association between dairy consumption and the risk of diabetes: A prospective cohort study from the China Health and Nutrition Survey
Source: Front Nutr. 2022 Sep 26;9:997636. doi: 10.3389/fnut.2022.997636 (PMC9550167; doi:10.3389/fnut.2022.997636)
Supplement: Supplementary file 1 [file Table_1.DOCX]

Supplementary Material

# Supplementary Data

The datasets were analyzed for this study can be found in the CHNS database: https://www.cpc.unc.edu/projects/china

# Supplementary Figures and Tables

**Table 1.** **HRs (95% CIs) of diabetes risk according to dairy consumption.**

|  | No Consumption | 0.1-100/Day | p | >100g/ Day | p |
| --- | --- | --- | --- | --- | --- |
| All participants | |  |  |  |  |
| Case/n | 327/12,357 | 44/2,194 |  | 19/945 |  |
| Model 1 | 1.00 (Reference) | 0.75 [0.55, 1.04] | 0.086 | 1.52 [0.95, 2.43] | 0.075 |
| Model 2 | 1.00 (Reference) | 0.53 [0.38, 0.73] | <0.001 | 0.96 [0.58, 1.59] | 0.868 |
| Model 3 | 1.00 (Reference) | 0.62 [0.44, 0.89] | 0.008 | 1.52 [0.88, 2.63] | 0.127 |
| Male |  |  |  |  |  |
| Case/n | 164/5,994 | 22/980 |  | 8/393 |  |
| Model 1 | 1.00 (Reference) | 0.82 [0.52, 1.27] | 0.369 | 1.46 [0.71, 2.98] | 0.299 |
| Model2 | 1.00 (Reference) | 0.50 [0.31, 0.80] | 0.003 | 0.82 [0.38, 1.74] | 0.596 |
| Model 3 | 1.00 (Reference) | 0.61 [0.38, 1.00] | 0.050 | 1.32 [0.60, 2.93] | 0.487 |
| Female |  |  |  |  |  |
| Case/n | 163/6,363 | 22/1214 |  | 11/552 |  |
| Model 1 | 1.00 (Reference) | 0.71 [0.46, 1.11] | 0.137 | 1.60 [0.86, 2.96] | 0.138 |
| Model 2 | 1.00 (Reference) | 0.55 [0.34, 0.88] | 0.013 | 1.11 [0.56, 2.19] | 0.773 |
| Model 3 | 1.00 (Reference) | 0.66 [0.40, 1.09] | 0.103 | 1.60 [0.75, 3.44] | 0.225 |
